# Nonlinear mixed effects dose response modeling in high throughput drug screens: application to melanoma cell line analysis

**DOI:** 10.18632/oncotarget.23495

**Published:** 2017-12-15

**Authors:** Kuan-Fu Ding, Emanuel F. Petricoin, Darren Finlay, Hongwei Yin, William P.D. Hendricks, Chris Sereduk, Jeffrey Kiefer, Aleksandar Sekulic, Patricia M. LoRusso, Kristiina Vuori, Jeffrey M. Trent, Nicholas J. Schork

**Affiliations:** ^1^ J. Craig Venter Institute, La Jolla, CA, USA; ^2^ University of California, San Diego, CA, USA; ^3^ George Mason University, Fairfax, VA, USA; ^4^ The Translational Genomics Research Institute, Phoenix, AZ, USA; ^5^ Sanford Burnham Prebys Medical Discovery Institute, La Jolla, CA, USA; ^6^ Yale University, New Haven, CT, USA

**Keywords:** high throughput drug screen, nonlinear mixed effect models, bioinformatics, cancer, drug response

## Abstract

Cancer cell lines are often used in high throughput drug screens (HTS) to explore the relationship between cell line characteristics and responsiveness to different therapies. Many current analysis methods infer relationships by focusing on one aspect of cell line drug-specific dose-response curves (DRCs), the concentration causing 50% inhibition of a phenotypic endpoint (IC_50_). Such methods may overlook DRC features and do not simultaneously leverage information about drug response patterns across cell lines, potentially increasing false positive and negative rates in drug response associations. We consider the application of two methods, each rooted in nonlinear mixed effects (NLME) models, that test the relationship relationships between estimated cell line DRCs and factors that might mitigate response. Both methods leverage estimation and testing techniques that consider the simultaneous analysis of different cell lines to draw inferences about any one cell line. One of the methods is designed to provide an omnibus test of the differences between cell line DRCs that is not focused on any one aspect of the DRC (such as the IC_50_ value). We simulated different settings and compared the different methods on the simulated data. We also compared the proposed methods against traditional IC_50_-based methods using 40 melanoma cell lines whose transcriptomes, proteomes, and, importantly, BRAF and related mutation profiles were available. Ultimately, we find that the NLME-based methods are more robust, powerful and, for the omnibus test, more flexible, than traditional methods. Their application to the melanoma cell lines reveals insights into factors that may be clinically useful.

## INTRODUCTION

Cancers have proven notoriously difficult to treat because of their cellular complexity and heterogeneity, their ability to coopt a number of naturally-occurring molecular processes and factors to sustain their growth, their capacity to evade and adapt to treatments through a rapid evolution, and a general lack of available drugs that can combat different mechanisms contributing to their initiation and growth. The identification of specific factors and processes that reveal points of vulnerability in cancers should reveal therapeutic targets, but relevant strategies for doing so are far from trivial. In addition, there is now growing consensus that the factors contributing to the initiation and growth of a tumor are nuanced and often very individual-specific, so that the best way to treat cancer is to identify the potentially unique determinants of an individual's tumor and therapeutically target those determinants. Such “personalization” efforts for cancer treatments are receiving a great deal of attention [1, 2].

Strategies for identifying the very heterogeneous determinants of cancer that might be used in the tailoring therapies to an individual are varied. Many large-scale epidemiological and tumor characterization efforts, such as the TCGA initiative [3], have proven to be useful but suffer from quality control and inadequate sample size issues [4]. Strategies that consider testing different treatment regimens on individual patients until an effective therapy is found – which is often what happens spontaneously in the clinical care of patients refractory to initial treatments – is problematic from both biological and ethical standpoints, since purposely using a drug that might be known to be ineffective simply to test another drug is unethical. In addition, drugs have effects on tumors that change their composition, as noted, which could thwart the logic behind attempts to switch a patient to a pre-specified list of drugs [5].

Alternatives to large-scale epidemiological and focused clinical studies on individual patients to find ways of identifying connections between drugs and tumor characteristics include tumorgraft (or xenograft) models and tumor cell line-based studies. Implanting tumors in mice or other organisms and studying the engrafted tumor's response to drugs has been used to great effect, but depends critically on the tumor environment in mice mimicking that in humans, which is not often the case [6, 7]. Cell line based models derived from actual patient tumors have been used, also to great effect, for some time [8, 9]. They are relatively easy and inexpensive to perform and can be used in high-throughput drug screening protocols (HTS), making them especially attractive for gaining at least initial insights into what drugs might be most effective against cells making up a tumor. Cell line models do suffer from the problem of extrapolating their *in vitro* responses to actual *in vivo* settings [10], but their aforementioned ease of use and amenability to HTS makes them particularly attractive [11]. In addition, emerging strategies for creating cell lines using, e.g., induced pluripotent stem (iPS) cells, have added to their sophistication [12].

Unfortunately, the reproducibility of cell line-based HTS experiments have been called into question [13]. Differences in protocols used, the media within which the cells are suspended, the precise formulation of the drugs used in the screening, the origins of the cell lines used, among other items, can create differences in outcome of studies involving what are thought to be the same cell lines. It is arguable that many of these technical sources of variation in cell line-based HTS studies can be identified and possibly controlled for in well-designed experiments [14]. However, the analytical methods used to draw inferences about the relationship between a subset of cell lines’ observed dose-dependent responses to a drug and characteristics of those cell lines (e.g., their genomic, transcriptomic, or proteomic profiles) also play an important (and often overlooked) role in the identification of factors that might mitigate a tumor's response to a drug.

Traditional statistical methods for analyzing cell line-based HTS data involve fitting (often sigmoidal) dose response curves (DRCs) to each cell line's dose response data in isolation and then extracting a single estimated parameter, the concentration causing 50% inhibition (i.e., the “IC_50_”) of a phenotypic endpoint, such as cell growth, from those curves [13, 15, 16]. These IC_50_ values are then tested for association with other factors, like the expression levels of genes measured on those cell lines, to identify markers of response. Analyzing each cell line and drug combination in isolation is highly problematic since it ignores variation exhibited across the cell lines that might inform the response profile of any single cell line. Leveraging variation across all the cell lines when making claims about drug responsive in any single cell line can reduce noise and lead to more reliable inferences.

In this light, we considered the use of non-linear mixed effects (NLME) models in analyzing HTS dose-response data, which considers variation across the data on all or subsets of the cell lines in order to estimate and assign a metric of drug responsiveness to each cell line. We considered two different NLME-based tests to compare against traditional methods. Both tests considered the evaluation of the cell lines collectively when estimating IC_50_ values and other DRC-related parameters. One test focuses exclusively on the IC_50_ values estimated from the cell lines simultaneously and hence is similar to traditional methods that focus on IC_50_ values, whereas the other test leverages a Likelihood Ratio (LR) formulation of an omnibus test in order to detect differences between DRC profiles among cell lines subsets and is thus theoretically much more flexible than IC_50_-based tests since it can capture differences between the DRCs among subgroups of cell lines beyond IC_50_ values.

**Figure 1:**
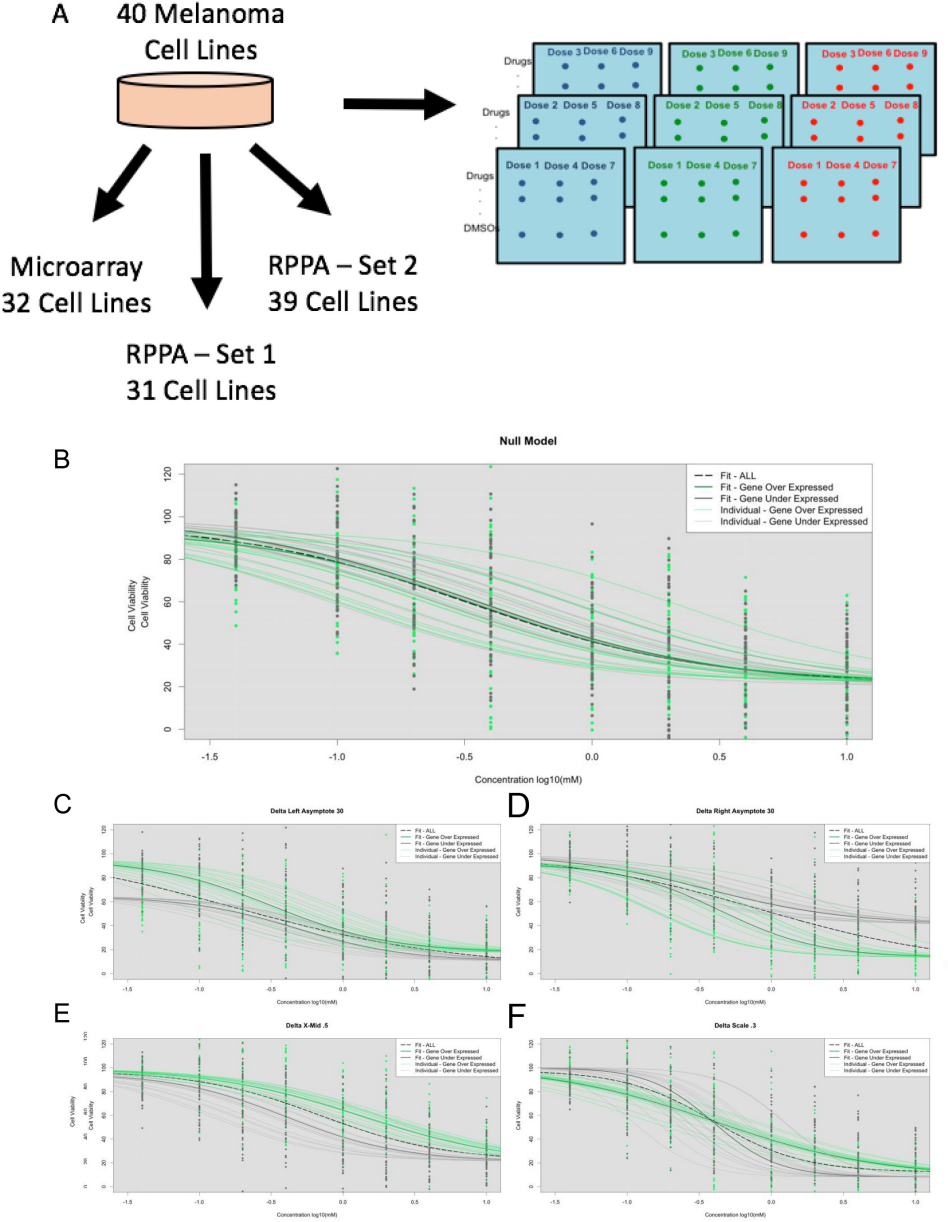
(**A**) Experimental design with melanoma cell lines processed with HTS, whole genome microarray genechip, and RPPA. We performed simulations under the HTS under the null model – no influence from GEX (**B**) and under the alternative model for left asymptote differences (**C**), right asymptote differences (**D**), X-Mid differences (**E**), Scale differences (**F**), and a combination of the parameters.

We applied the proposed NLME-based models and tests to 40 available melanoma cells lines with genomic, transcriptomic, and proteomic profiles available that had been screened against 120 drugs (Figure 1A). We compared the results obtained with the traditional IC_50_-based data analytic methods to the proposed NLME-based methods. We also considered analyses with the available Cancer Cell Line Encyclopedia [1, 8] (CCLE) data sets, and performed simulation studies to explore the relative advantages of each method in different situations that go beyond those focusing on IC_50_ values. We find that the proposed NLME-based tests are more powerful, sensitive and, for the omnibus test, flexible than traditional methods. In fact, the LR-based NLME test can be used to uncover associations among various factors collected on the cell lines, such as gene expression levels, and drug responsiveness in a very robust and compelling way, and that are simply beyond the reach of analyses focusing exclusively on IC_50_ values. Both proposed NLME-based methods may reveal factors mitigating cancer drug response that could inform “personalized” or targeted therapeutic approaches to cancer in actual patient care. We also consider a few extensions of the proposed methods in the Discussion section.

## RESULTS

### Simulation studies

As noted, we simulated DRCs assuming triplicate cell line evaluations (to mimic are actual data) for sample sizes of 34, 50, and 100 triplicate cell lines (Figures 1B-1F). Based on the results of the analyses of this simulated data using each method (i.e., traditional tests using individual cell line-based IC_50_ values, the IC_50_ NLME test and Omnibus LR NLME tests) we calculated the false positive rate or type I error rate (i.e., using the null model simulation settings) and the false negative rate, power or type II error rate (using the alternative model simulation settings). Power analysis revealed that 100 simulations for these analyses would suffice (Supplementary Figure 1). For the type I error rate, we found that each of the three association tests produced results close to the expected value of 5% false positives, regardless of the number of cell line samples (Table 1). However, the simulation results for the Omnibus LR NLME tests suggested it performed better in nearly every scenario (with the exception of the largest right asymptote changes, where the IC_50_ NLME association tests performed better (Table 2). We note that for Table two, the three tests can seem to be unequally sensitive to differences in DRCs that exhibit differences in parameters beyond the IC_50_, (since we fixed one of the parameters and averaged the estimates of the power estimates when the other parameters were allowed to vary). This was to be expected, since the traditional IC_50_ test estimates IC_50_ values for each cell line independently and then explores the relationship between those estimated IC_50_ values and some other factor on those cell lines. Thus, it essentially ignores several important cell line properties. The NMLE IC_50_ test, although focused on the IC_50_ values, is influenced by the other DRC parameter differences between the two groups since it estimates parameters for the groups using all the data. Therefore, it is able to detect differences in DRCs beyond IC_50_ values.

**Table 1:** Simulation-based power to detect differences between DRCs between two groups assuming different features about the groups’ DRCs

Group 1 Parameters					Group 2 Parameters					Power		
Simulated CLs	Left	Right	XMid	Scale	Simulated CLs	Left	Right	XMid	Scale	Trad IC50	NLME IC50	LLR Test
50	100	10	–0.5	–0.5	50	100	10	–0.5	–0.5	0.06	0.11	0.01
100	100	10	–0.5	–0.5	100	100	10	–0.5	–0.5	0.04	0.06	0.04
50	100	10	–0.5	–0.5	50	100	10	–0.4	–0.5	0.07	0.18	0.02
50	100	10	–0.5	–0.5	50	100	10	–0.25	–0.5	0.16	0.66	0.24
50	100	10	–0.5	–0.5	50	100	10	0	–0.5	0.39	1.00	0.90
50	100	10	–0.5	–0.5	50	100	10	–0.5	–0.4	0.00	0.06	0.49
50	100	10	–0.5	–0.5	50	100	10	–0.5	–0.2	0.06	0.08	0.86
50	100	10	–0.5	–0.5	50	100	20	–0.5	–0.5	0.02	0.34	0.81
50	100	10	–0.5	–0.5	50	100	30	–0.5	–0.5	0.07	0.90	0.99
50	100	10	–0.5	–0.5	50	100	40	–0.5	–0.5	0.04	1.00	0.88
50	100	10	–0.5	–0.5	50	90	10	–0.5	–0.5	0.05	0.34	0.69
50	100	10	–0.5	–0.5	50	80	10	–0.5	–0.5	0.04	0.86	0.98
50	100	10	–0.5	–0.5	50	70	10	–0.5	–0.5	0.02	1.00	0.98
100	100	10	–0.5	–0.5	100	100	10	–0.4	–0.5	0.09	0.20	0.07
100	100	10	–0.5	–0.5	100	100	10	–0.25	–0.5	0.29	0.96	0.63
100	100	10	–0.5	–0.5	100	100	10	–0.5	–0.4	0.05	0.07	0.84
100	100	10	–0.5	–0.5	100	100	40	–0.5	–0.5	0.09	1.00	1.00
100	100	10	–0.5	–0.5	100	90	10	–0.5	–0.5	0.05	0.63	0.99
100	100	10	–0.5	–0.5	100	80	10	–0.5	–0.5	0.09	1.00	1.00
100	100	10	–0.5	–0.5	100	70	10	–0.5	–0.5	0.04	1.00	1.00

Key: Values shown present the number of cell lines (CLs), the left asymptote, right asymptote, X-Mid, and Scale for each simulation. Power results were calculated using tests based on traditionally estimated IC_50_ values, NLME estimated IC_50_, and the Omnibus LLR tests. In each simulation, one group was held constant with the following parameters. The values highlighted in red denote differences in the assumed parameter values for the two groups. Note that the first two rows are simulations under the null hypothesis (i.e., no difference in parameter settings between the two groups).

**Table 2:** The mean and SD of power observed when a single parameter is fixed between two groups and all other parameters are varied in one of the groups (see methods section)

Fixed Parameter	Varied Parameters	Trad IC_50_	NLME IC_50_	LLR
Left Asym = 100	Right Asym, X-Mid, Scale	0.12 +/– 0.11	0.73 +/– 0.35	0.77 +/– 0.24
Left Asym = 90	Right Asym, X-Mid, Scale	0.13 +/– 0.10	0.61 +/– 0.38	0.73 +/– 0.24
Left Asym = 80	Right Asym, X-Mid, Scale	0.11 +/– 0.08	0.42 +/– 0.32	0.85 +/– 0.07
Left Asym = 70	Right Asym, X-Mid, Scale	0.09 +/– 0.08	0.49 +/– 0.33	0.83 +/– 0.09
Right Asym = 10	Left Asym, X-Mid, Scale	0.12 +/– 0.10	0.41 +/– 0.33	0.69 +/– 0.29
Right Asym = 20	Left Asym, X-Mid, Scale	0.12 +/– 0.10	0.46 +/– 0.33	0.78 +/– 0.14
Right Asym = 30	Left Asym, X-Mid, Scale	0.12 +/– 0.10	0.61 +/– 0.36	0.87 +/– 0.08
Right Asym = 40	Left Asym, X-Mid, Scale	0.11 +/– 0.08	0.93 +/– 0.13	0.86 +/– 0.08
X-Mid = -0.5	Right Asym, Left Asym, Scale	0.05 +/– 0.02	0.48 +/– 0.34	0.79 +/– 0.21
X-Mid = -0.4	Right Asym, Left Asym, Scale	0.05 +/– 0.02	0.47 +/– 0.35	0.76 +/– 0.23
X-Mid = -0.25	Right Asym, Left Asym, Scale	0.12 +/– 0.03	0.59 +/– 0.37	0.80 +/– 0.20
X-Mid = 0	Right Asym, Left Asym, Scale	0.28 +/– 0.05	0.79 +/– 0.32	0.82 +/– 0.10
Scale = -0.5	Right Asym, Left Asym, X-Mid	0.12 +/– 0.09	0.62 +/– 0.36	0.74 +/– 0.20
Scale = -0.4	Right Asym, Left Asym, X-Mid	0.12 +/– 0.11	0.58 +/– 0.35	0.76 +/– 0.24
Scale = -0.2	Right Asym, Left Asym, X-Mid	0.12 +/– 0.09	0.53 +/– 0.38	0.88 +/– 0.07

When exploring specific settings, the association tests using traditionally derived IC_50_ values (i.e., that are estimated independently from each cell line) exhibited a type 1 error rate consistent with the null model (e.g., 0.05) in cases where differences in DRCs were simulated that involved changes in left asymptote, right asymptote, and scale (Table 1). Note that in Table one, the values highlighted in red denote differences in assumed parameter values between the two groups. Moreover, we do find that the traditional IC_50_ association tests performed significantly poorer when the x-mid parameters were fixed (i.e., x-mid were the same in both groups; first two rows of Table 1). This was expected since, as noted, the IC_50_ association tests are designed to detect differences between changes in the X-Mid (i.e., estimated IC_50_ values) parameters (Table 1). However, in both the IC_50_ NLME test and Omnibus LR NLME tests, there is a marked improvement in the power to detect the DRC differences (Table 1). Although there was a slight improvement in the simulated settings where differences in x-mid values were assumed, interestingly the traditionally-derived IC_50_ association tests (i.e., where each cell line's DRC x-mid or estimated IC_50_ value is estimated independently) exhibited much less power.

We compared the statistical power of each of the tests using paired two-sample *t*-tests and found that the traditionally derived IC_50_ association tests performed worse than the NLME (paired 2-sample *t*-tests statistics = 11, pval < 2.2e-16) and LLR tests (paired 2-sample *t*-tests statistics = 27, pval < 2.2e-16). Even upon increasing the number of sample sizes to 50 and 100 cell lines, the Omnibus LR NLME tests had greater statistical power than IC_50_ NLME and traditional IC_50_-based association tests (Supplementary Table 1, Supplementary Figure 2). As noted, when the x-mid parameter is held constant, i.e. the x-mid is the same in both group, then the traditional IC_50_ association tests are close to the assumed Type I error rate, as expected (Table 1). We also assessed the residuals for the NLME curve fits and found the residuals to follow a normal distribution (Supplementary Figure 3). Ultimately, we observed that the Omnibus LR NLME tests exhibited the greatest power to detect differences in the dose response curves, without sacrificing type I errors or generating many false positive associations. However, it also proved to be the most computationally demanding method for association tests.

### Comparing the different methods on the gene expression data

**Figure 2:**
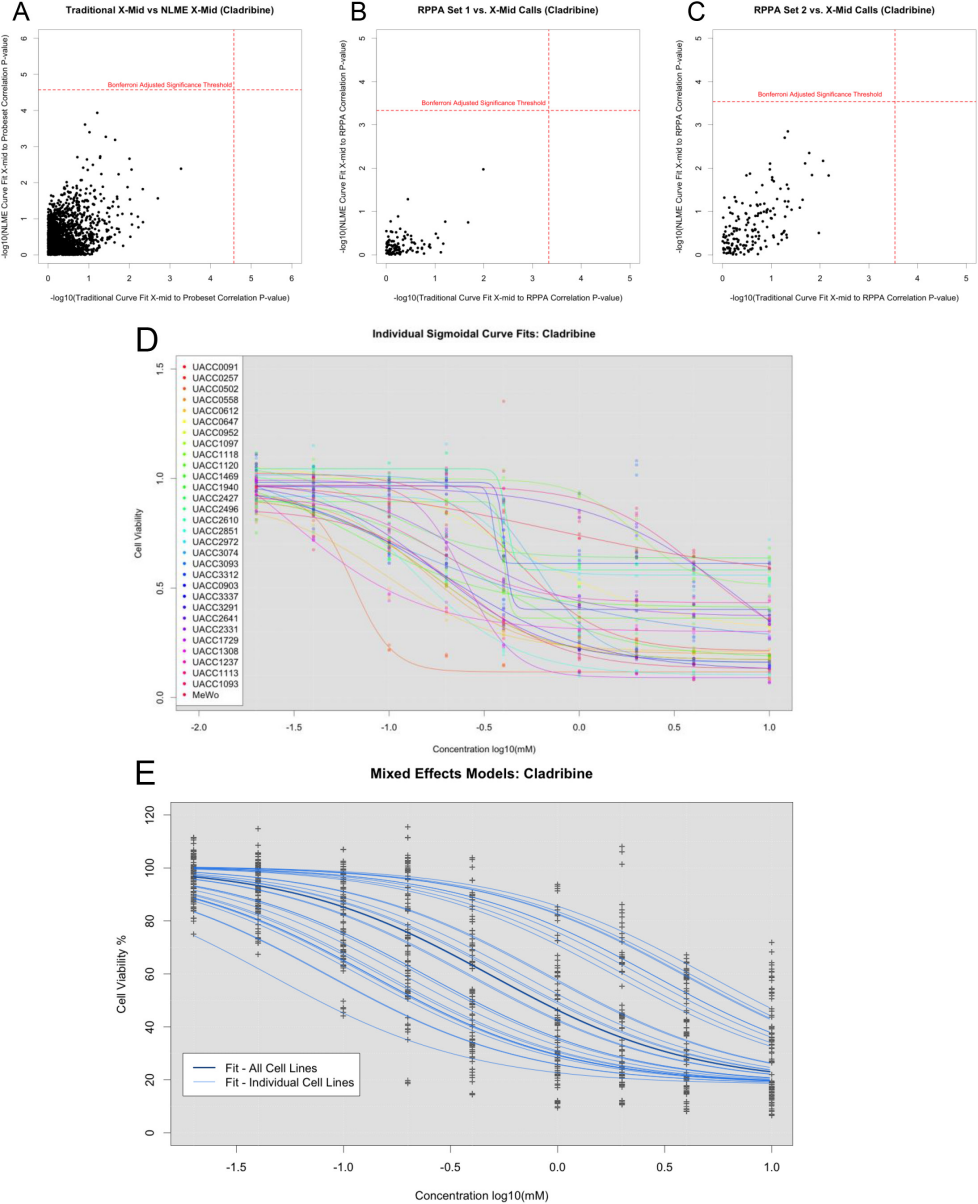
Differences observed between using traditionally called IC_50_, NLME called IC_50_, and LLR tests (**A**) -log10*p*-values for associations between probe set expressions and traditionally called IC_50_ or NLME called IC_50_. (**B**) -log10*p*-values for associations between RPPA set 1 expression and traditionally called IC50 or NLME called IC50. (**C**) -log10*p*-values for associations between RPPA set 2 expression and traditionally called IC_50_ or NLME called IC_50_. (**D**) Dose response curves fit individually for Cladribine. (**E**) Dose response curves leveraging information across cell lines, which balances inter- and intra- cell line variability.

To assess the properties of association tests based on traditionally estimated IC_50_ (x-mid) values against the IC_50_ NLME test, we performed association tests using drugs with NLME curve fits to cell lines that had at least one cell line with cell viability less than 20%. Since a subset of the drugs in our study target the BRAF-mediated pathway, we suspect that differential drug response may be observed based on the BRAF associated genes’ expression patterns. However, based on analyses of these 15 drugs, no BRAF associated genes were significantly associated when using IC_50_ NLME test, and only four significant associations were found with traditional IC_50_ calls: HSPA9 (adjusted *p*-value < 0.027) and KSR1 (adjusted *p*-value < 0.028) with Vorinostat; RAF1 (adjusted *p*-value < 0.007) and HSPA9 (adjusted *p*-value < 0.03) with Crizotinib. Scatterplots comparing these correlations highlighted the differences in the nature of the associations between the traditional IC_50_ and IC_50_ NLME-based methods (Figure 2A). Although there was a general positive correlation, we observed a great deal of variation observed when using the two different curve fit methods.

### Comparing the different methods on the RPPA data

Similar to the gene expression analysis, we compared the associations between proteins and drug responses defined by the traditional IC_50_ and the IC_50_ NLME-based tests across the cell lines (Supplementary Table 3). As with the results observed for the gene expression correlation analysis, we found that using different methods for obtaining the IC_50_ parameters yielded different sets of genes associated with drug response (Figure 2B–2C). Across all drugs, we found only significant RPPA associations when using IC_50_ NLME tests (Caspase 6 (cleaved Asp162) with the drug Thioguanine Bonferroni adjusted *p*-value = 0.026). However, using traditional methods, we found seven significant associations in the first RPPA set and eleven significant associations in the second RPPA set. Interestingly, when we applied simulation models, the NLME models yielded less false positives. Thus, we suspect that the increased associations identified using traditional methods may be false positives rather than true hits.

### Assessing the omnibus LR NLME test

**Figure 3:**
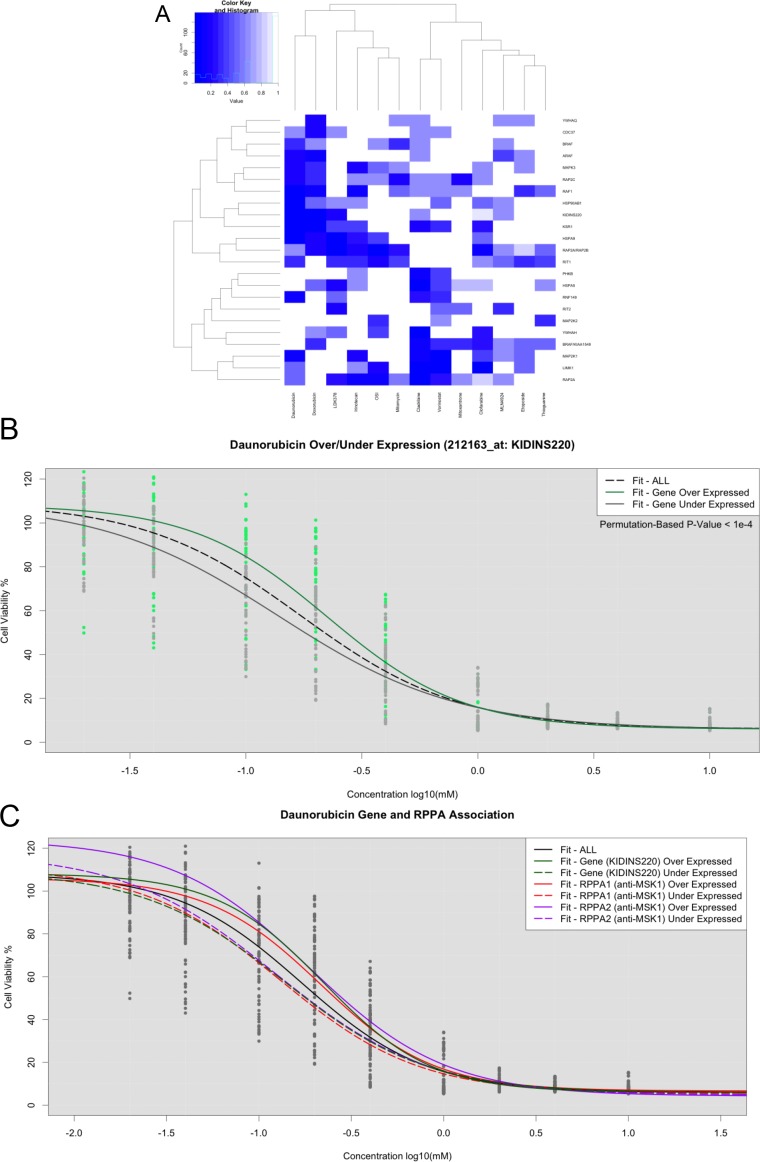
LLR tests for assessing significant gene expression association in BRAF-related genes (**A**) Heatmap of *p*-values across 15 drugs. (**B**) Dose response curves fit within KIDINS220 over-expression (Green) and under-expression (Grey). (**C**) Dose response curves fit in over- and under-expressed phosphoproteins with strong associations.

To test whether gene expression was associated with overall drug response profiles and not just IC_50_ values, we stratified cell lines based on their gene expression levels for those genes in the BRAF pathway. For each gene, the samples were either labeled as over-expressed or under-expressed based on the median intensity level across all cell lines. LLRs were calculated for each gene and drug combination, and permutation-based tests were performed to assess statistical significance (Figure 3A-3B, Supplementary Table 3). Notably, only a small fraction of the differentially expressed genes were significantly associated with drug response. This result is particularly noticeable in the heatmaps representing the differential effects (Figure 3A). Specifically, the more significant gene associations appear to be limited to Daunaribicin, Doxorubicin, Vorinostat, and Cladribine (Figure 3A). To account for multiple hypothesis testing, we increased the permutations to a total of 9,999 for those with potentially significant *p*-values based on the initial Omnibus LR NLME tests. We found a single gene association significant beyond multiple hypothesis testing corrections (KIDDINS220 with Daunorubicin, *p*-value < 1e-4). However, there were several other cases that yielded strong (but not beyond multiple hypothesis testing correction thresholds) association between gene expression and drug response (Figure 3C).

### Assessing the consistency between the RPPA and gene expression analyses

To investigate potential relationships between gene and protein expression, we used un-adjusted NLME association *p*-values associated with each drug to identify proteins encoded by specific genes that were also seemingly associated with drug response. Using this strategy, many genes exhibited gene expression levels and their corresponding protein levels that were significantly associated with drug response. After consistencies between the GEX and RPPA analyses were found, we mined molecule-interaction databases to identify a biological basis for the observed results. Admittedly, this was not an exhaustive search, but we found several compelling cases to support efforts to combine insights from transcriptomics and proteomics using the NLME models and LLR tests. For Daunorubicin, we found the increased expression of phosphorylated MSK1 (anti-MSK1: phosphor S360) conferred resistance, whereas the increased expression of phosphorylated PKC (Phospho-PKCα/βII: Thr638) and PKCα (anti-PKC: Ser657) were associated with sensitivity to the drug (Figure 3C, Supplementary Figures 5–6). In another compelling example, over-expression of MAPK3 was a significant predictor of response to Cladibrine. Furthermore, we observed that cell lines responsive to Cladibrine also had lower expression in the protein pBAD S112, which is known to be associated with the BCL pathway (Supplementary Figures 7–8). Similarly, we found that over-expression of the KSR1 gene was associated with response to the drug LDK378 (Supplementary Figure 9). KSR1 and FKHR have many physical interactions or are co-expressed with many of the same genes (Supplementary Figure 10).

### Analysis of the CCLE data with NLME-based models

To explore whether or not the manner in which the IC_50_ values were estimated had an impact on the identification of relationships between drug response and gene expression values, we fit NLME models across all the CCLE cell lines for each available drug and compared the results of these NLME-assigned x-mid (IC_50_) values against the available IC_50_ values computed by the CCLE research team (Supplementary Figure 11). Although we observed a general concordance between the two IC_50_ estimates, we found that the methods yielded different values across the cell lines. Additionally, fitting NLME curves across cancer types allowed us to assess each drug's response profile within cancer types. For example, we identified AZD-6244 as a candidate drug in melanoma since it had a more significant NLME curve fit relative to other cancer types.

### Including mutation status as random effect in CCLE data

**Figure 4:**
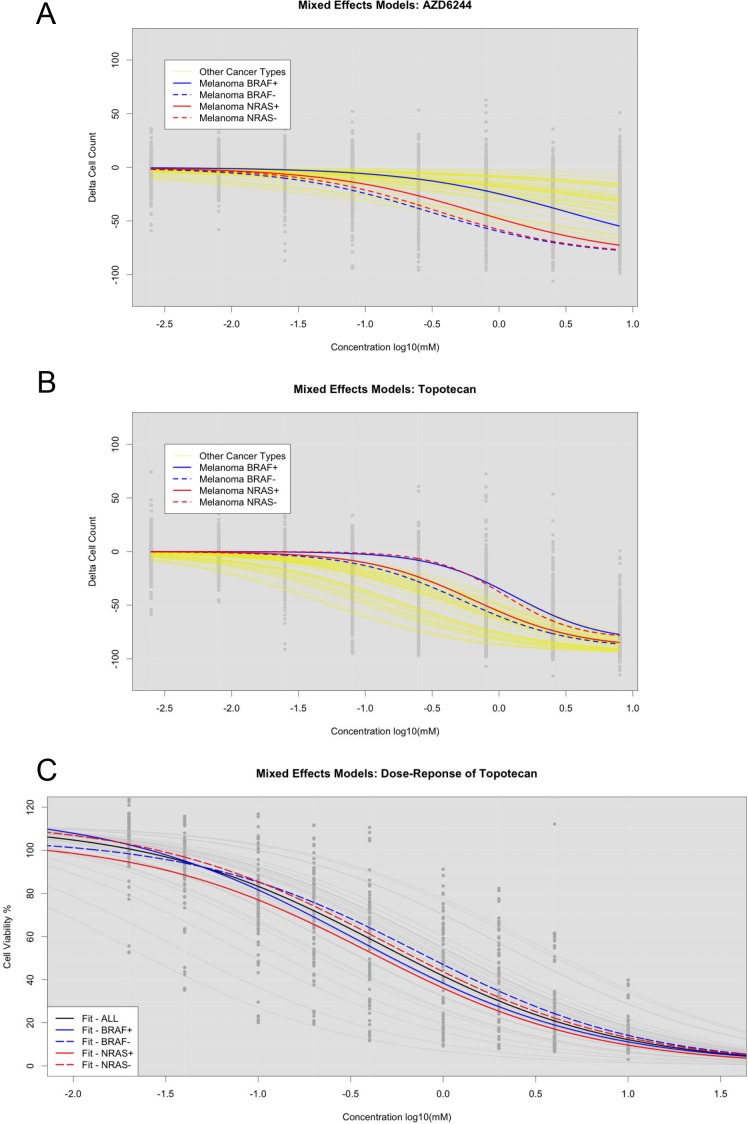
LLR tests for assessing significant stratification of cell lines with or without mutations in CCLE and SU2C cell lines (**A**) Assessment of AZD6244 in CCLE across all cell lines (Black), melanoma BRAF+ (Solid Blue), and melanoma BRAF- (Dotted Blue). (**B**) Same as A, but with Topotecan. (**C**) Assessment of Topotecan in SU2C across all cell lines (Black), BRAF+ (Solid Blue), BRAF- (Dotted Blue), NRAS+ (Solid Red), and NRAS- (Dotted Red).

Fitting NLME curves within groups of cell lines stratified by mutation status could provide more accurate IC_50_ assessments if mutation status impacts drug responsiveness. Based on our NLME curve fits to the CCLE cell lines, we found that, overall, melanoma cell lines were more sensitive to the drug AZD-6244 than other cancer types. To assess the impact of incorporating mutation status into NLME curve fits, we stratified the cell lines into BRAF mutant verse BRAF wild type cell lines. Although the mutation status did not impact curve fits for most CCLE drugs, we found that the BRAF wild type samples were much more resistant to AZD-6244 (Figure 4A). NRAS mutations were also associated with response to AZD-6244. However, melanoma cell lines with NRAS mutations appeared to have a greater sensitivity than those with BRAF mutations (Figure 4A).

To assess concordance between our UACC melanoma cell lines and the CCLE melanoma cell lines, we compared NLME curve fits by subdividing the cell lines by BRAF and NRAS mutation status in both data sets. We found cases where mutation status had an opposite impact on curve fits between the two data sets – though this is likely due to differences in cell lines used. For example, for the drug Topotecan, we found that NRAS mutations led to greater sensitivity and BRAF mutations led to greater resistance in the CCLE cell lines (Figure 4B) compared to the UACC cell lines. However, in our UACC cell lines, both NRAS and BRAF mutations led to greater sensitivity to the Topotecan (Figure 4C). This suggests that while the mutation status could help improve NLME curve fits, careful data analyses should be considered. In this particular case, we suggest incorporating the NRAS mutation status, but omitting the BRAF mutation status, in fitting NLME curves for Topotecan in melanoma cell lines.

## DISCUSSION

HTS studies using tumor cells lines can illuminate the relationship between drug responsiveness and various factors collected on those cell lines (like gene expression levels, mutation status, etc.). Such relationships can be exploited clinically to “personalize” cancer treatments. However, there have been recent concerns surrounding the reliability HTS studies using tumor cell lines [1, 2, 3, 8, 9, 10, 11]. One question about such screens concerns the analysis methods used to draw inferences about the relationships between cell line drug responses and characteristics of the cell lines. Traditional methods of measuring drug response in HTS consider cell lines in isolation and do not leverage information across all available cell lines used in the screen. To overcome this limitation, we considered NLME models to analyze multiple dose response curves simultaneously to account for the variability across the cell lines. Thus, instead of using single parameters extracted from individual dose response curves to assess associations, we use overall curve fits to a group of cell lines that accommodates association tests. The two tests we introduced, the IC_50_ NLME-based test and the Omnibus LR NLME test, each have advantages and disadvantages. The clear advantage of the Omnibus LR test is that it does not consider a single parameter in the analysis of DRCs, but rather assumes the overall shapes of the curves between two groups (we note that it would be easy to generalize the test to more than two groups) defined on an assumed associated cell line characteristic like gene expression level, are different.

We pursued simulation studies to assess false positive rates and the statistical power of NLME methods relative to traditional methods. Our simulations showed that NLME-based tests, in particular the proposed Omnibus LR NLME test, and are more powerful than standard tests. However, these simulations are very computationally expensive, especially for the Omnibus LR NLME test. Due to the computational burden, we suggest that if adequate computational resources are not available, then the use of more efficient tests that still leverage NLME models, such as the IC_50_ NLME test, can be used.

We applied the NLME-based tests in order to see if they could identify differential gene expression and protein level associations with drug response in more powerful and compelling ways than traditional individual DRC IC_50_-based analytical methods. We found evidence for many associations that could not be attributed to chance. Upon mining gene and protein interaction databases, we observed that many of the associations we observed several were consistent with previously published data. For example, we found that the differential expression of the MAPK3 gene stratified melanoma cell lines into Cladribine responders and non-responders, which is consistent with what is known about the mechanism of action of Cladribine. Based on this finding, and in conjunction with the interrogation of the DGIdb database, we found that kinase inhibitors 5-Iodotubercidin and Purvalanol are likely drug candidates for these cell lines.

Our analyses and observations suggest that NLME-based models for fitting and analyzing DRCs could complement current HTS strategies and infrastructure. However, these NLME methods require, as noted, a fairly heavy computational burden, especially if one wants to rely on permutation-based methods for obtaining *p*-values for the Omnibus LR NLME test, and may not be optimal in every context; e.g., when one wants to search the entire HTS space for gene by drug interactions which could be very computationally demanding. While traditional association tests require the least computational resources and could be run locally, the NLME association tests require slightly more computational resources (approximately 4 hours and 8 GB of RAM per drug tested) and the Omnibus LR NLME test required even more computational resources (greater than 24 hours and 8GB of RAM per drug tested). In an ideal setting, every association among genes, proteins, and drugs could be explored. Given the computational burden, we opted for a biologically-guided search (i.e., searching for BRAF-related genetic associations in melanoma) that ultimately led to compelling associations between genes and drug responses. The use of NLME-based analysis approaches that we document here can be extended to test associations between other important factors (e.g., Copy Number Variations, DNA methylation status, Single Nucleotide Polymorphisms, etc.) and drug response.

In addition, there are some very useful statistical modeling extensions of the proposed methods that could be exploited. First, bootstrap methods could be used to obtain standard errors for the parameter estimates [23]. This may be computational challenging unless the focus is on a particular drug or small set of drugs. Certainly when evaluating the power and accuracy of the proposed tests, pursuing as many simulations as possible is appropriate. We only pursued 100 simulations for our studies, but considered many different settings, and have no reason to believe the overall trends we observed would change, but only their precision, if we pursued more simulations. Second, if the interest is in the analysis of a single drug or a set of drugs, confidence intervals for the dose-response curves themselves, rather than an of the parameters governing them, can be obtained [24]. Third, our models assumed normal error distributions; however, the residual distributions could be assessed. Again, this may be difficult if thousands of drugs are tested in different settings, but would be useful for further evaluating promising drug candidates. Fourth, for the proposed omnibus test, one could explore which parameters are driving a difference between two groups if an overall difference is found. This could be achieved by fixing all but one of the parameters to be equivalent between the two groups and the re-evaluating the LR test systematically. Fifth, as an alternative to the omnibus test in which group difference are of focus, one could conceivably include a factor hypothesized to be associated with response into an NLME model as a covariate and then compare it to a model without that factor included. The power and robustness of this approach could be compared with our strategy of comparing groups based on the factor in question. Ultimately, we believe that the NLME approach to analysis of HTS dose-response data is flexible, robust and powerful enough to be used routinely.

## MATERIALS AND METHODS

We made use of a number of resources in our evaluation of different methods for analyzing cell line-based DRC data generated from HTS data. We briefly describe these resources before providing greater detail about the construction and execution of the two proposed NLME-based models and tests.

### Cell lines

Thirty-three melanoma cell lines from the University of Arizona Cancer Center (UACC) repository were used for our analyses. Additionally, the SK-MEL-2 cell line was received directly from Memorial Sloan Kettering Cancer Center through the Cancer Cell Line Encyclopedia (CCLE) repository and MeWo cell line from the Developmental Therapeutics Program's NCI-60 repository. The Translational Genomics Research Institute (TGen) had access to all other UACC-cell lines through the UACC [14]. All cell lines were of low passage number. Cells were maintained according to the manufacturer's or collaborator's instructions. All media used to grow and harvest the cell lines had 10% FBS and 1% AA added to final growth media, and all cell lines were banked at low passages in multiple aliquots and liquid nitrogen stocks to reduce risk of phenotypic and genetic drift. All cells were cultured for less than three months before reinitiating culture from the frozen stock, were routinely inspected for identity by morphology and growth curve analysis and validated to be free of mycoplasma and contaminants.

### Drug screening

Each of the 40 cell lines was used in a nine-point (i.e., concentration/dose) HTS study (drug concentrations: 0.02, 0.04, 0.1, 0.2, 0.4, 1.0, 2.0, 4.0, and 10.0 μM) across 120 drugs (Figure 1A, Supplementary Table 1) at the Sanford Burnham Prebys Medical Discovery Institute (SBPMDI or SBP in the sequel). Drugs were spotted on 384-well clear bottom tissue culture treated plates (Greiner Bio-One, #781098) using an Echo Liquid Handler (Labcyte Inc., Sunnyvale, CA) such that addition of 25Ls of cells (100 k cells/mL in RPMI +10% FBS +Pen./ Strep./ Glut., Omega Scientific, Tarzana, CA) resulted in the above-described final drug concentrations and 2.5 k cells/well. Upon plating, the cells were gently spun down at 1k rpm for one minute and incubated with drugs for 96 hours at 37°C in a standard tissue culture incubator. After this time course, plates were allowed to equilibrate to room temperature for 30 minutes before 10 μLs per well of freshly prepared CellTiterGlo reagent (G7571, Promega Corp., Madison WI) were added. Samples were incubated for ten minutes with gentle agitation (100 rpm) before luminescence was read on a BioTek Synergy2 plate reader using Gen5 software (BioTek, Winooski, VT). Each plate was assayed in triplicate and included 24 vehicle-only DMSO controls.

### Gene expression assays

The cell line samples were subjected to nucleic acid extraction, verification, amplification, and hybridization per the protocol regarding the use of the Affymetrix HG-U133 plus 2.0 arrays (54,675 probesets, Affymetrix, Santa Clara, CA). Affymetrix HG-U133 Plus 2.0 microarrays were normalized (background adjustment, interquartile normalization, and median polish) using robust multichip averaging [17] in R.

### Phosphoproteomic assays

Cell lysates from the cell lines were printed in triplicate onto nitrocellulose-coated slides (Grace Bio-Lab, Bend, OR) using a 2470 Aushon arrayer equipped with 185 mm pins (Aushon BioSystems, Burlington, MA) and subjected to Reverse Phase Protein Array (RPPA) analysis at George Washington University. For quality control purposes, standard curves were printed on each array along with the samples. To quantify the amount of total protein in each sample, selected arrays were stained with Sypro Ruby staining solution (Molecular Probes, Eugene, OR) pursuant to manufacturer recommendation. Before proceeding with immunostaining, arrays were first incubated in Reblot Stripping solution (Chemicon, Temecula, CA) for 15 minutes, washed twice with PBS (Life Technologies, Carlsbad, CA) and incubated in I-Block (Tropix, Bedord, MA) for one hour. Using an automatic system (Dako Cytomation, Carpinteria, CA), arrays were then incubated with commercially available 3% hydrogen peroxidase solution, avidin-biotin blocking system, and protein block (Dako Cytomation, Carpinteria, CA). The expression/activation level of a panel of FDA-approved and/or under investigation drug targets and their downstream effectors was measured using a single primary antibody targeting the protein and phosphorylation site of interest. Each antibody used on the array was previously validated using western blot to confirm its specificity. A commercially available tyramide-based avidin/biotin amplification system (CSA; Dako Cytomation, Carpinteria, CA) and fluorescent detection (LI-COR Biosciences, Lincoln, NE) were used to quantify the amount of protein present in each sample. Antibody stained slides were scanned using the Tecan laser scanner (Tecan PowerScanner Tecan group Ltd., Männedorf, Switzerland). Images were analyzed with MicroVigene 5.1.0.0 (Vigenetech, Carlisle, MA) as previously described [18]. Because the output of the RPPA platform is quantitative, the intensity values obtained from the analysis were reported on a continuous variable.

### CCLE data set

To provide another comparison and evaluation of the performance of different analysis methods, we downloaded and processed raw CEL files from the CCLE. We obtained gene expression profiles and genome-wide mutation calls for melanoma cell lines from the CCLE web portal.

### Traditional IC_50_ DRC analysis

For each of the 40 cell line's 9-point dose response data generated for each of 120 drugs from the melanoma drug screen, we fit DRCs using a traditional four parameter sigmoidal curve. The parameters governing these curves were: left asymptote of the curve, right asymptote of the curve, midpoint of the curve (referred to as the “x-mid” value), and a scale parameter [19]. All analyses, including those involving the proposed NLME models and simulation studies described below were performed on the San Diego Supercomputer Center (TSCC). To fit DRCs to each cell of the melanoma cell line's 9-concentration DRC data, we used the nplr [19] package in R. We used a similar DRC analyses for the CCLE data and simulated data described below. To determine whether the IC_50_ values were significantly associated with the gene expression, we used Pearson correlation tests with the paired gene expression and estimated IC_50_ values across the cell lines.

### NLME-based analyses

As noted, we considered two different NLME-based models and tests for the DRC data, each assuming the same four parameter sigmoidal curve as in the traditional IC_50_ analyses discussed in the previous section. For the first NLME-based model, which we will refer to as the “IC_50_ NLME” model, we fit four parameter sigmoidal curves to all the cell lines simultaneously allowing for variation in IC_50_ values across the cell lines. We leveraged the lme4 [20] package in R with fixed effects for each of the four parameters in the sigmoidal curve and an additional random effect for the “x-mid” (i.e., an estimate of the IC_50_) parameter while using the cell line as a grouping factor to do so. Note that the IC_50_ NLME analysis model produces IC_50_ values for each cell line that can be used in Pearson correlation tests to determine their association with other factors collected on those cell lines, such as gene expression levels, but just estimated simultaneously with the other cell lines.

For the second test, which we will refer to as the “Omnibus LR NLME” model and test, we fit four parameter sigmoidal curves to stratified subsets of the cell lines based on grouping factors, such as the cell lines expressing a gene above and below the median gene expression value (real or simulated, depending on the data set) or cell lines with and without a mutation. The likelihoods obtained from each group's curve fits were then used to calculate a log-likelihood ratio statistic comparing the DRCs between the two groups as:

$$LLR=-2*LL_{all}+2*(LL_{Gene+}+LL_{Gene-})$$

Note that since the Omnibus LR NLME test does not focus on a single parameter, like the x-mid or IC_50_ value, it can in theory identify differences between DRCs obtained from two (or more) groups that could involve the left or right asymptote, the scale parameter, the x-mid parameter or any combination of these [21]. To determine the significance of the observed LLR statistic, 100 permutations were performed for, e.g., the over and under expressed status for each cell line. For each permuted sample, we calculated LLRs and for each drug *i* and each permutation *j*, we calculated C_ij_ as:

$$C_{ij}=\{\begin{array}{c} 1,if\;LLR_{ij}>LLR_{i}\\ 0,\text{otherwsie} \end{array}$$

Then, based on these permutations, for n total permutations, the *p*-value for the observed stratification was:

$$p_{i}=\frac{1+\sum_{j=1}^{n}C_{ij}}{n+1}$$

To save computational resources, when association test *p*-values were going to exceed 0.05 based on the number of permutations we set (i.e., they were not significant and could not achieve significance given the number of permutations), we stopped the permutations for that setting and proceeded to the next test setting. Additionally, we employed adaptive permutations [22]; i.e., for the associations that were significant at a 0.05 *p*-value, we increased the number of permutations to meet multiple hypothesis testing criterion (e.g., we increased permutations to 10,000 when testing for associations between 23 genes and 15 drug tests).

We explored the utility of the proposed Omnibus LR NLME tests relative to the traditional tests in a number of scenarios with the melanoma cell lines. We computed statistics and *p*-values using the RPPA values stratifying the call lines at the median intensity value. We also ran tests on all BRAF associated genes for which we had gene expression data and the top five most significant phosphoproteins using Pearson correlation tests for the IC_50_ NLME and traditional IC_50_ test. As a check on the robustness of the analyses, we also assessed significance on five random analytes for each drug. Bonferroni corrections were used to account for multiple comparisons given the number of genes, proteins and mutations we tested for association with drug responses across the cell lines. For the analyses involving the CCLE data set, we compared BRAF mutant and wild type cell lines, and compared the results against analyses using the IC_50_ values provided the Broad Institute for the CCLE data set [8]. We consider extensions of the proposed analytical methods in the Discussion section.

### Simulation studies

We simulated High Throughput Screen (HTS) nine-point Dose Response Curves (DRCs) data assuming a four parameter sigmoidal model. We note that each simulation required heavy computational resources (30–120+ hours and 8GB of RAM per simulated parameter set). We assumed that the standard deviation of the response for each concentration followed that of the observed drugs’ standard deviations calculated from the actual melanoma cell line data. We simulated null models in which there was no assumed relationship between a hypothetical factor and drug response. In addition, we simulated cell line DRCs assuming sample sizes of 34, 50, and 100 in triplicate with a left asymptote of 100, right asymptote of 10, scale of -0.5, and x-mid values following a normal distribution with mean -0.5 and standard deviation of 0.3 (i.e., ∼N(-0.5, 0.3)) (Figure 1B). These values were determined based on averages from analyses involving all the actual melanoma samples. We then considered simulations in which half of the cell lines were randomly assigned as over-expressing a gene with a distribution of N(8, 1) and the other half were assigned as expressing that gene to a lesser degree with a distribution of N (3, 1). Based on these simulated parameters and standard deviation at each concentration, DRCS were simulated for each cell line. For each set of parameters, 100 simulations were performed and subsequent association tests were applied as follows: *t*-test of over-expressers vs. under-expressers with traditionally called IC_50_, *t*-test of over-expressers vs. under-expressers with NLME model-derived IC_50_ values, and the Omnibus LR test with the NLME-fitted DRCs. Keep in mind that differences in the test results between the IC_50_ values from the traditional analysis and the IC_50_ NLME called IC_50_ values reflect the influence of analyzing all the cell lines together rather than individually.

To evaluate the power of the association tests by exploring the impact of changes in DRCs not associated with IC_50_ values, we simulated dose response curves under different alternative models. In these simulations, under-expressed genes were simulated with different sigmoidal curve parameters described in Figure 1C-E. Note that in some settings, we averaged the power of the simulations in which one parameter was fixed but the others were allowed to vary to determine how easily the three tests could detect differences in DRCs between two groups that were not exclusively due to differences in IC_50_ values. To create realistic simulations, we explored various combinations (153 of the possible 192 combinations, approximately 80%) of the parameters and performed 100 simulations for each set of parameters.

## SUPPLEMENTARY MATERIALS FIGURES AND TABLES


