# Supplementary material for: Nonlinear mixed effects dose response modeling in high throughput drug screens: application to melanoma cell line analysis
Source: Oncotarget. 2017 Dec 15;9(4):5044–57. doi: 10.18632/oncotarget.23495 (PMC5797032; doi:10.18632/oncotarget.23495)
Supplement: Supplementary file 1 [file oncotarget-09-5044-s001.pdf]

Nonlinear mixed effects dose response modeling in high throughput drug screens: application to melanoma cell line analysis

SUPPLEMENTARY MATERIALS

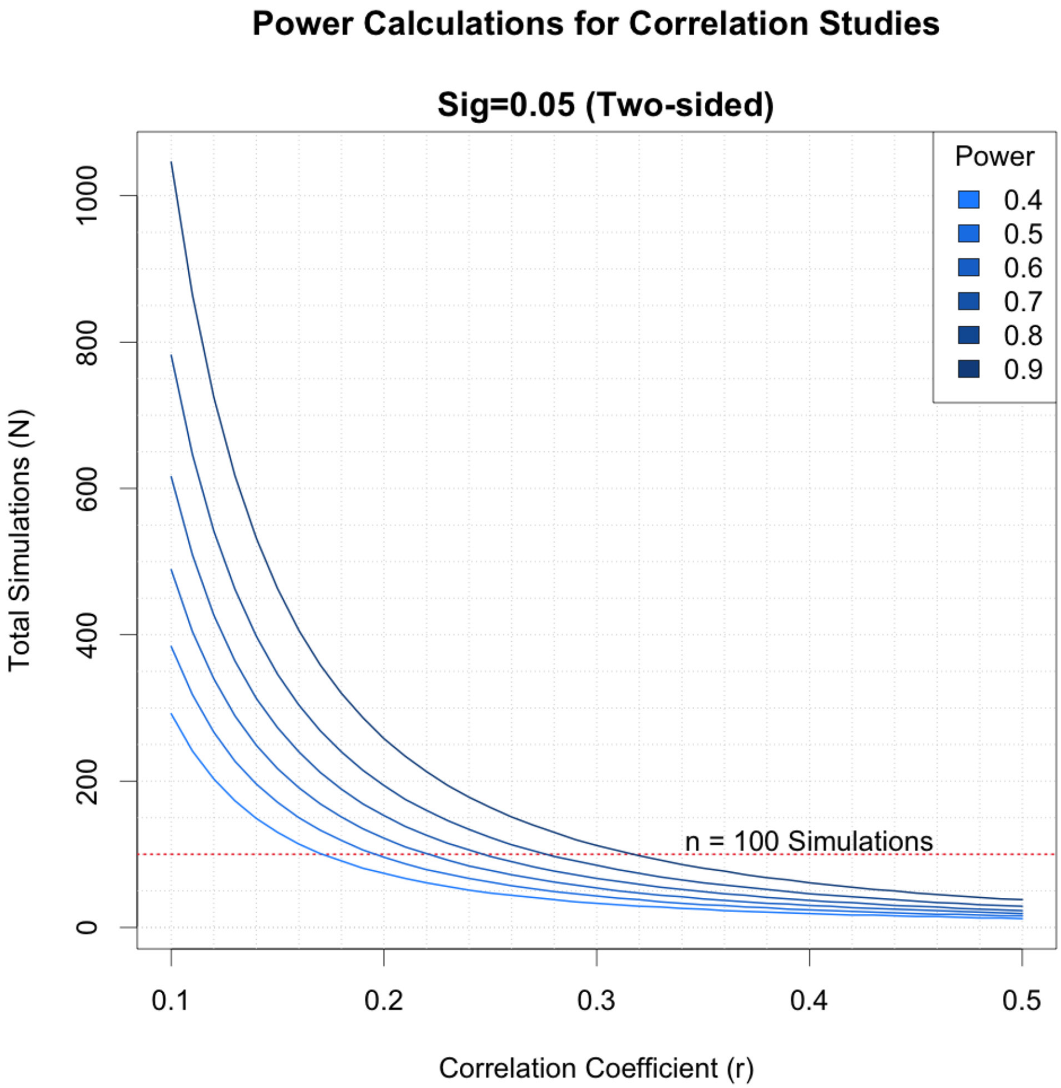

Supplementary Figure 1: Power analysis for simulations. Based on the observed correlations from initial parameter simulations (cor > .5), 100 simulations should suffice.

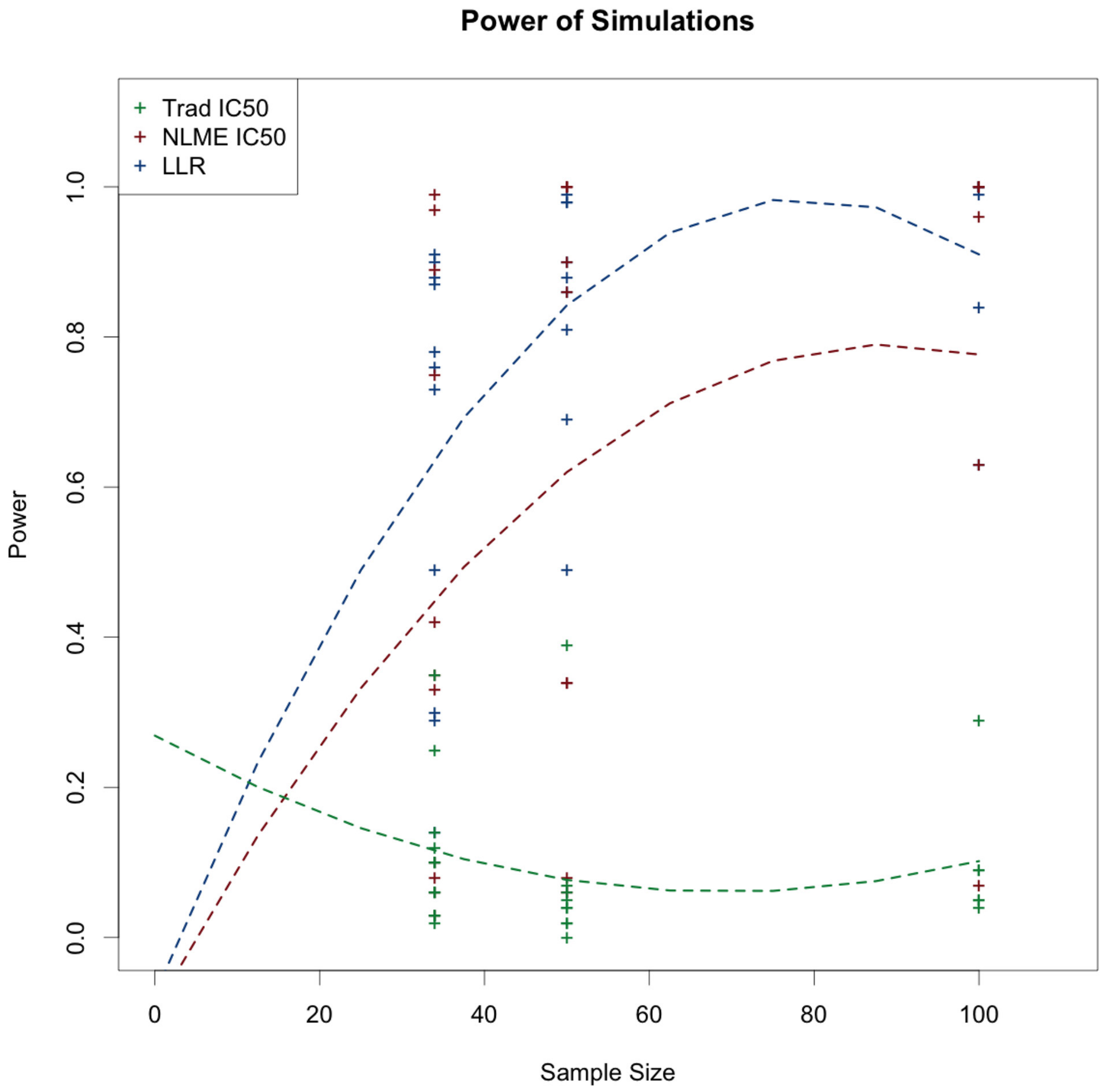

Supplementary Figure 2: Simulation-based power does improve with increased sample size in both the NLME called IC_{50} associations and in the LLR associations. Statistical power in traditionally called IC_{50} associations tests yielded the worst results.

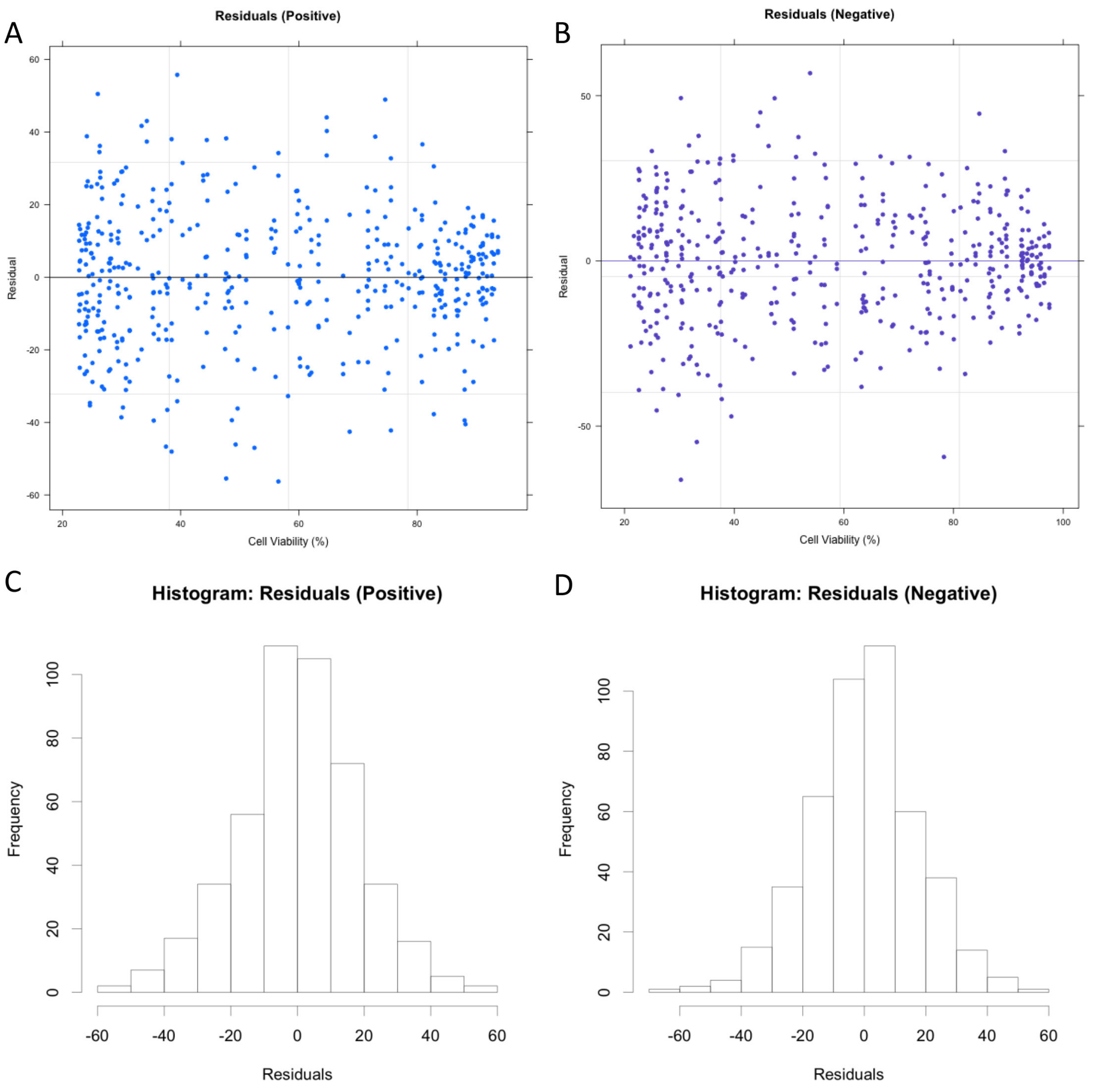

Supplementary Figure 3: Residuals for curve fits based on simulated over (**A, C**) or under (**B, D**)-expressed genes. A/B are scatterplots of the residuals and C/D are histograms of the residuals.

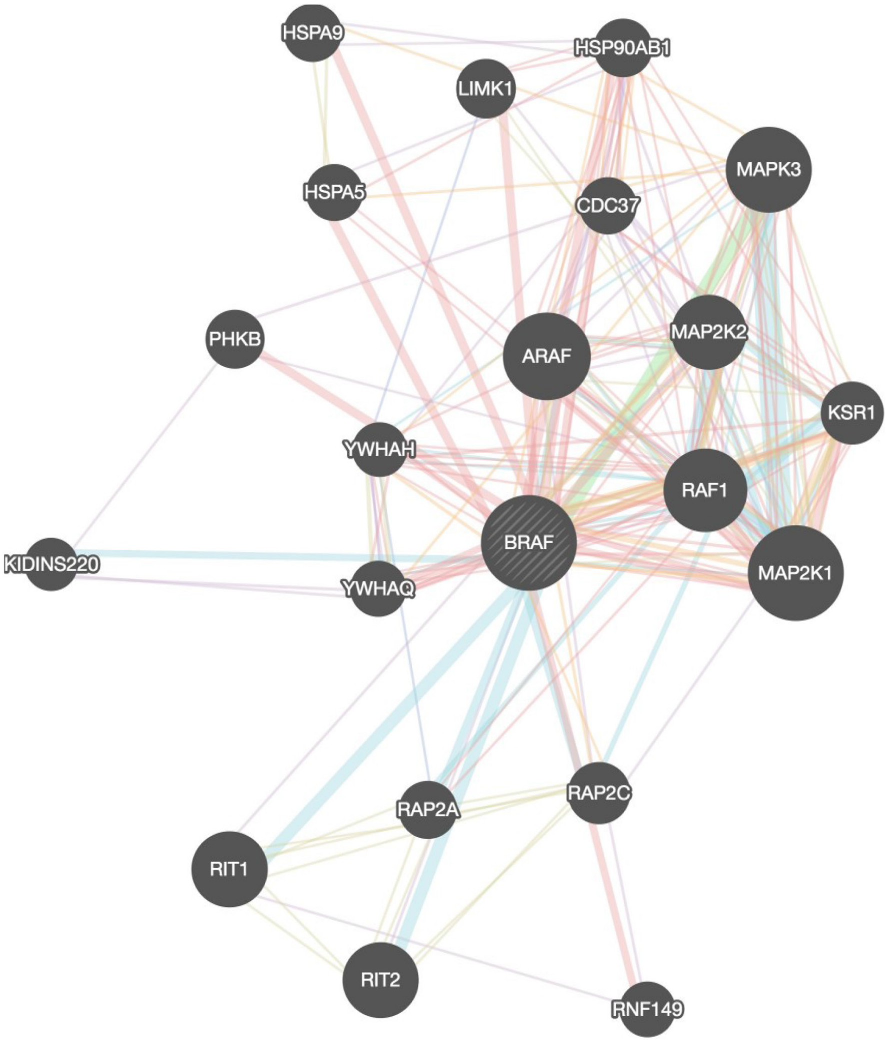

Supplementary Figure 4: GeneMANIA BRAF-related network with K = 20. Key: Pink = Physical interactions; Purple = Co-expression; Orange = Predicted; Light Blue = Pathway; Dark Blue = Co-localization; Green = Genetic interactions; Yellow = Shared protein domain; and size of circle is proportional to the number of connections.

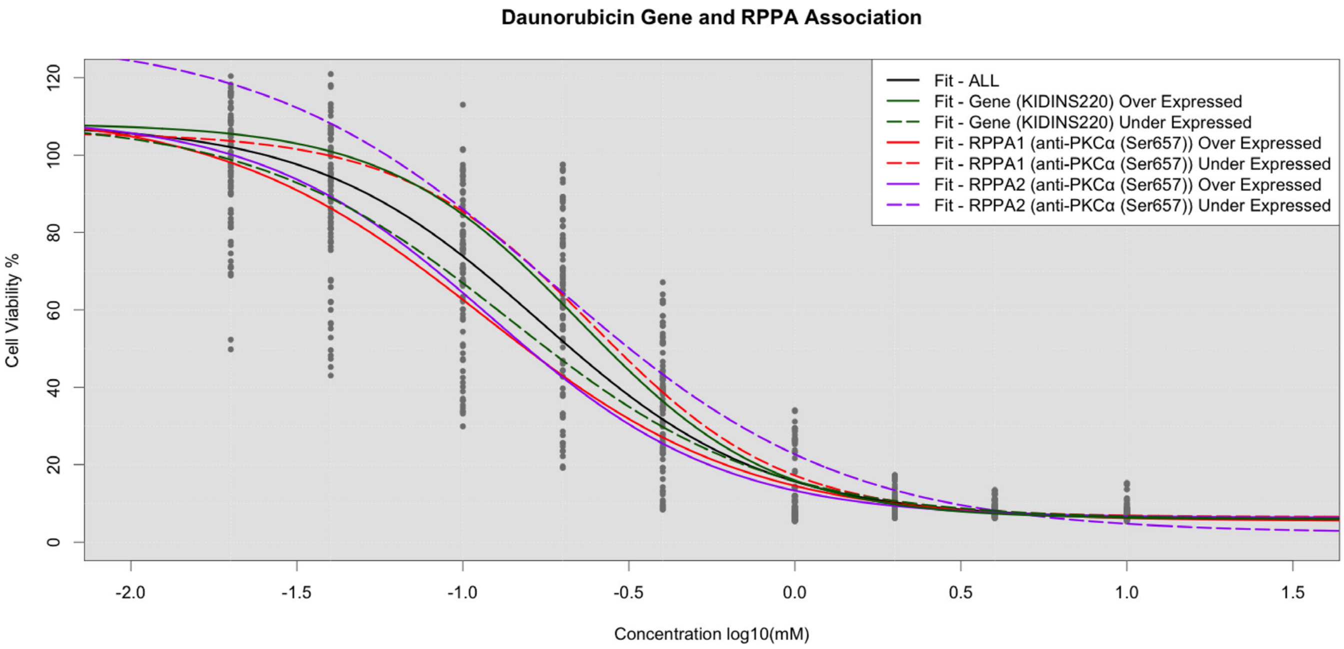

Supplementary Figure 5: Application of LLR tests to identify concordant RPPA (Phospho-PKCα/ βII (Thr638)) and Gene expression (KIDINS220) that stratify Dauanorubicin dose response curves.

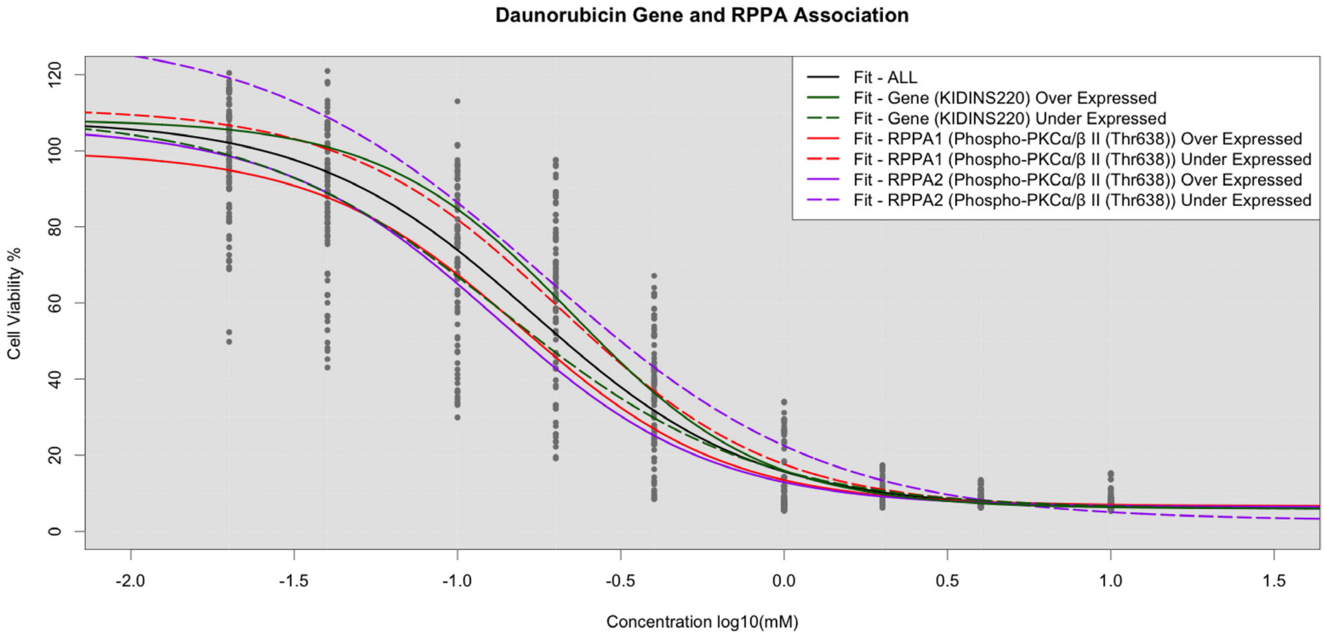

Supplementary Figure 6: Application of LLR tests to identify concordant RPPA (anti-PKCα (Ser657)) and Gene expression (KIDINS220) that stratify Dauanorubicin dose response curves.

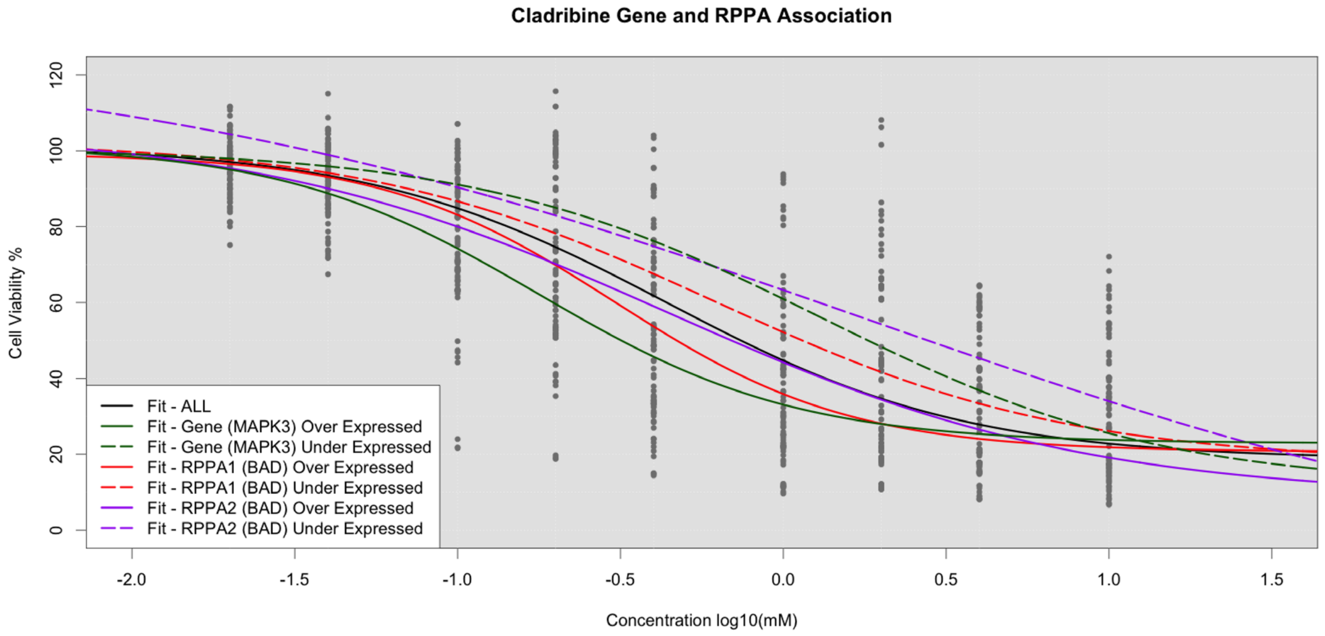

Supplementary Figure 7: Application of LLR tests to identify concordant RPPA (pBAD) and Gene expression (MAPK3) that stratify Cladribine dose response curves.

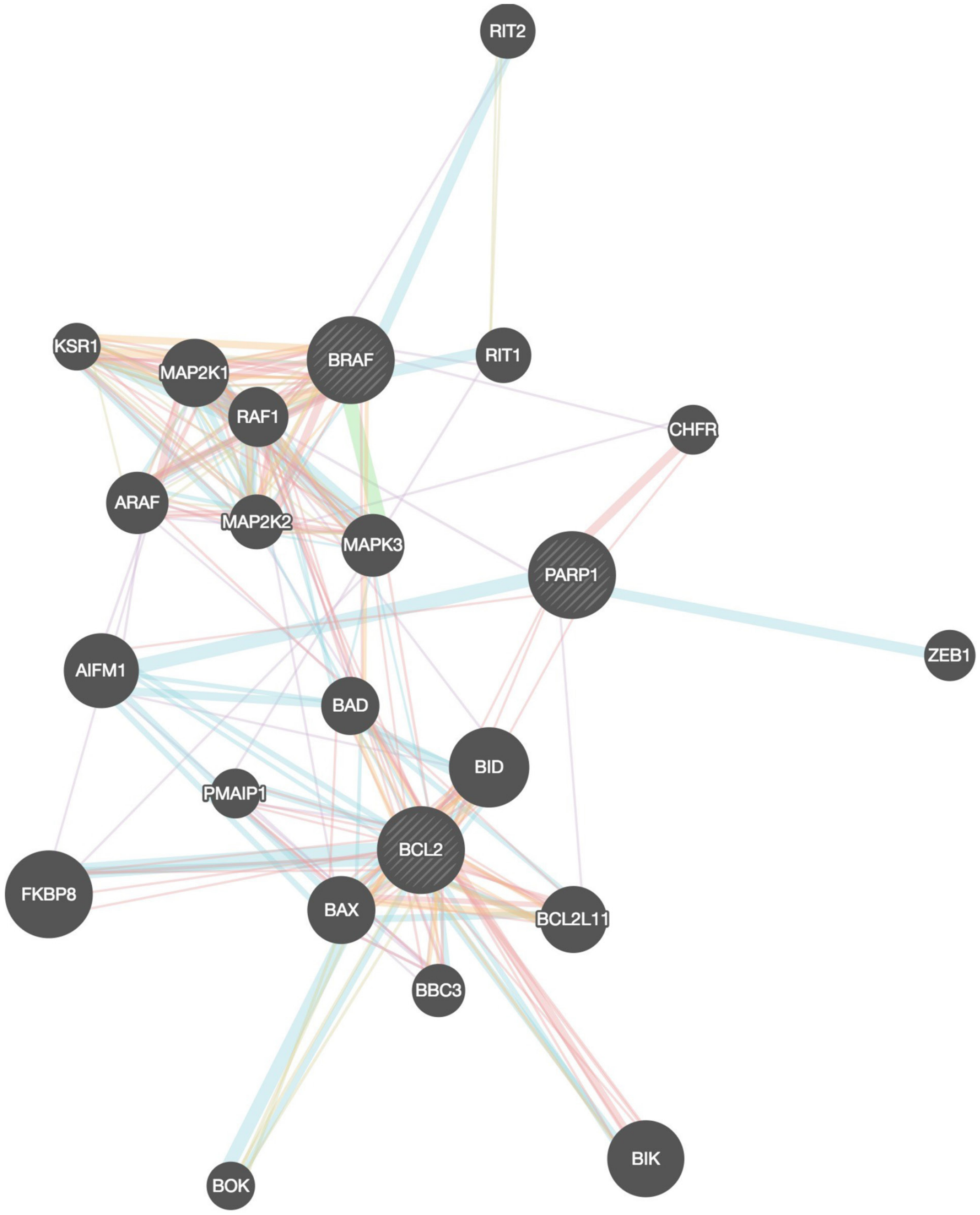

Supplementary Figure 8: GeneMANIA pathway analysis using BRAF and BCL2 showing the network connection of MAPK3 and pBAD.

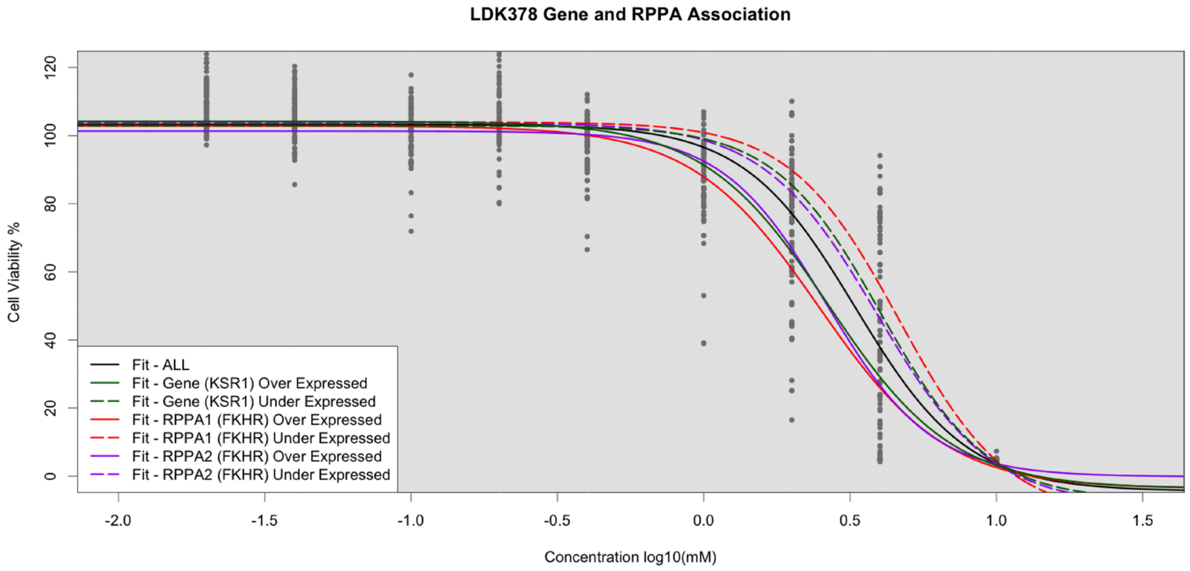

Supplementary Figure 9: Application of LLR tests to identify concordant RPPA (FKHR) and Gene expression (KSR1) that stratify LDK378 dose response curves.

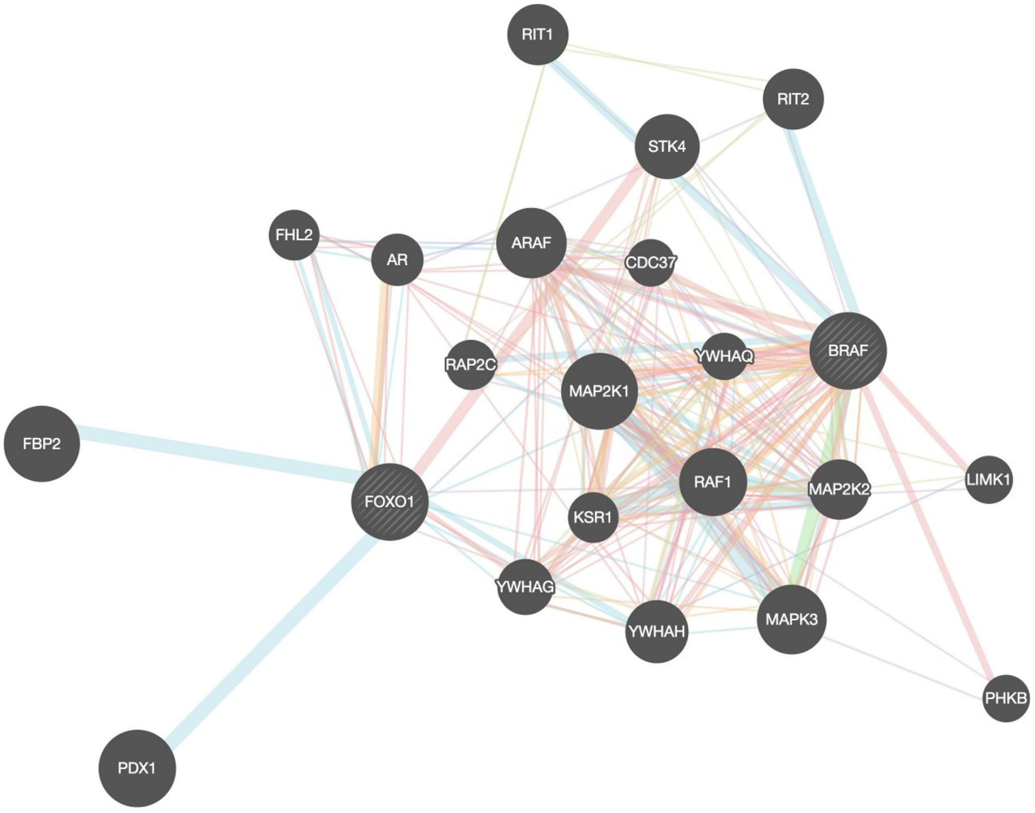

Supplementary Figure 10: GeneMANIA pathway analysis using BRAF and BCL2 showing the network connection of FOXO1 (FKHR) and KSR1.

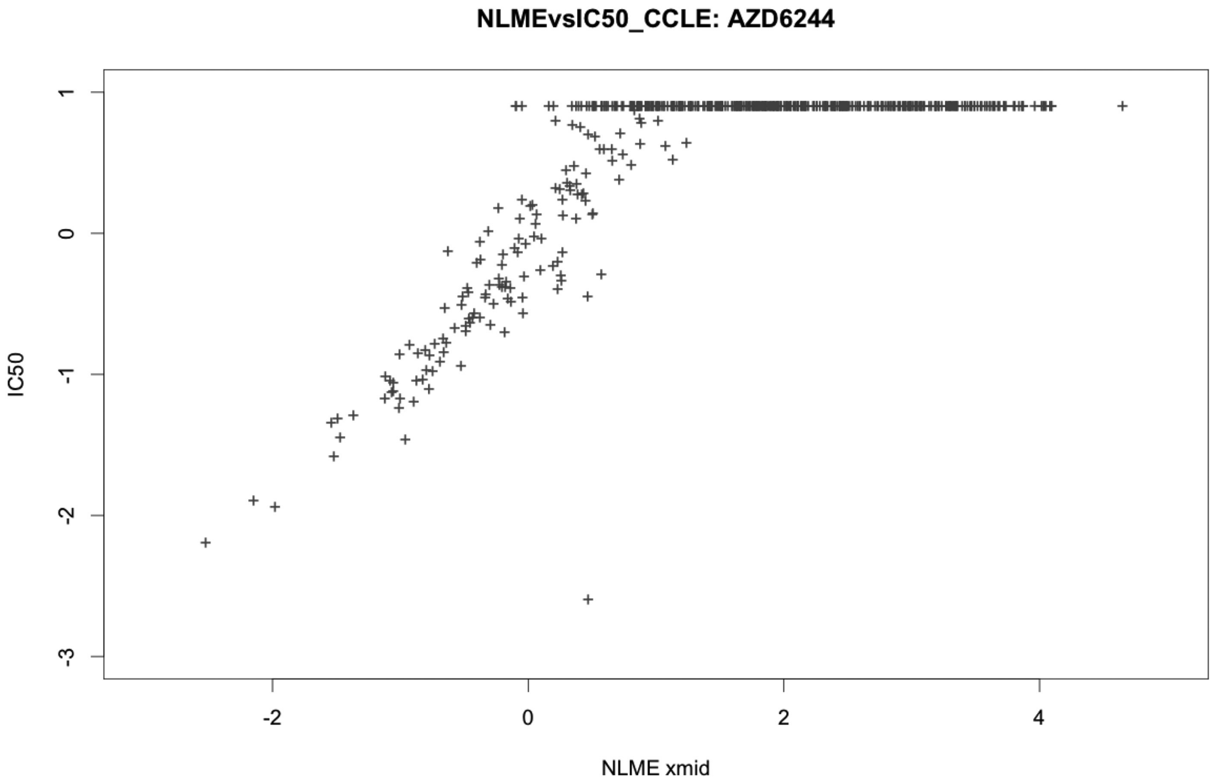

Supplementary Figure 11: Comparing the effects of estimating IC_{50} values (uM) with NLME models and those of traditional methods. Although overall correlation is observed, there are some clear variations.

Supplementary Table 1: Table of cell lines available in the SU2C study

SU2C Cell Lines	Nine Point HTS	GEX	RPPA Set 1	RPPA Set 2
A375	Y	N	N	Y
HEMnLP	Y	Y	N	N
MeWo	Y	Y	N	Y
SKMEL113	Y	N	N	Y
SKMEL119	Y	N	N	Y
SKMEL2	Y	Y	N	Y
SKMEL21	Y	N	N	Y
SKMEL217	Y	N	N	Y
UACC0091	Y	Y	Y	Y
UACC0257	Y	Y	Y	Y
UACC0502	Y	Y	Y	Y
UACC0558	Y	Y	Y	Y
UACC0612	Y	Y	Y	Y
UACC0647	Y	Y	Y	Y
UACC0903	Y	Y	Y	Y
UACC0952	Y	Y	Y	Y
UACC1093	Y	Y	N	Y
UACC1097	Y	Y	Y	Y
UACC1113	Y	Y	Y	Y
UACC1118	Y	Y	Y	Y
UACC1120	Y	Y	Y	Y
UACC1237	Y	Y	Y	Y
UACC1265	N	Y	N	N
UACC1308	Y	Y	Y	Y
UACC1469	Y	Y	Y	Y
UACC1649	Y	N	N	Y
UACC1729	Y	Y	Y	Y
UACC1940	Y	Y	Y	Y
UACC2331	Y	Y	Y	Y
UACC2427	Y	Y	Y	Y
UACC2496	Y	Y	Y	Y
UACC2610	Y	Y	Y	Y
UACC2641	Y	Y	Y	Y
UACC2851	Y	Y	Y	Y
UACC2972	Y	Y	Y	Y
UACC2994	Y	N	N	Y
UACC3074	Y	Y	Y	Y
UACC3093	Y	Y	Y	Y
UACC3291	Y	Y	Y	Y
UACC3312	Y	Y	Y	Y
UACC3337	Y	Y	Y	Y

HTS represents availability of high throughput drug screen, GEX represents availability of whole genome microarray genechips, and RPPA sets 1 and 2 represent the availability of Reverse Phase Protein Arrays.

Supplementary Table 2: Total number of significant associations based on Traditionally called IC_{50} and NLME called IC_{50} vales

	Azacitidine	Doxorubicin	Cladribine	Vorinostat	Crizotinib	Thioguanine	Daunorubicin	OSI027
RPPA Set 1 Trad Sig	0	0	1	0	0	3	3	1
RPPA Set 1 NLME Sig	0	0	0	0	0	1	0	0
RPPA Set 2 Trad Sig	1	1	0	2	1	4	1	1
RPPA Set 2 NLME Sig	NA	0	0	0	0	NA	0	0

Supplementary Table 3: *P*-values for LLR tests stratifying gene expression at the median value

Probeset	Gene Name	Azacitidine	Doxorubicin	Cladribine	Vorinostat	Clofarabine	Etoposide	Thioguanine
221830_at	RAP2A	NA	1.000	0.010	0.250	0.866	1.000	1.000
212163_at	KIDINS220	NA	0.010	0.667	1.000	0.904	1.000	0.944
201244_s_at	RAF1	NA	0.091	0.667	0.667	1.000	0.286	0.500
200064_at	HSP90AB1	NA	0.500	1.000	0.500	0.500	1.000	1.000
200691_s_at	HSPA9	NA	0.167	1.000	1.000	0.500	0.941	1.000
213699_s_at	YWHAQ	NA	0.200	0.667	1.000	1.000	0.667	1.000
201020_at	YWHAH	1.000	0.667	0.051	1.000	0.222	1.000	NA
201895_at	ARAF	1.000	0.080	0.667	1.000	1.000	0.667	1.000
202424_at	MAP2K2	1.000	1.000	1.000	0.667	1.000	1.000	0.300
202670_at	MAP2K1	1.000	1.000	0.111	0.010	0.400	0.500	1.000
202738_s_at	PHKB	1.000	1.000	0.125	0.400	1.000	1.000	1.000
204357_s_at	LIMK1	NA	1.000	0.059	0.010	0.074	0.500	NA
206044_s_at	BRAF /// KIAA1549	NA	0.286	0.100	0.333	0.222	0.500	0.500
206984_s_at	RIT2	NA	1.000	1.000	0.500	1.000	1.000	1.000
209882_at	RIT1	NA	1.000	1.000	1.000	0.667	0.300	0.400
209953_s_at	CDC37	NA	0.143	0.667	0.667	1.000	1.000	1.000
211936_at	HSPA5	0.750	0.500	0.010	0.400	0.800	1.000	0.667
212046_x_at	MAPK3	NA	0.286	1.000	1.000	0.667	0.667	1.000
235252_at	KSR1	1.000	0.020	0.200	0.333	0.222	NA	1.000
214487_s_at	RAP2A /// RAP2B	0.667	0.154	1.000	1.000	0.111	0.852	0.500
218669_at	RAP2C	1.000	0.333	0.667	0.667	0.667	NA	1.000
225414_at	RNF149	NA	1.000	0.250	0.333	1.000	1.000	1.000
226391_at	BRAF	NA	0.667	0.667	1.000	1.000	NA	1.000

Probeset	Gene Name	Irinotecan	Mitoxantrone	Mitomycin	Daunorubicin	OSI	MLN4924	LDK378
221830_at	RAP2A	0.125	0.667	0.600	0.500	0.125	0.667	0.222
212163_at	KIDINS220	1.000	1.000	1.000	0.010	1.000	0.667	0.200
201244_s_at	RAF1	0.111	0.667	0.500	0.010	1.000	1.000	1.000
200064_at	HSP90AB1	0.667	1.000	1.000	0.067	1.000	0.667	0.667
200691_s_at	HSPA9	0.222	NA	NA	0.059	0.400	1.000	0.051
213699_s_at	YWHAQ	1.000	1.000	0.667	1.000	1.000	0.667	1.000
201020_at	YWHAH	1.000	NA	1.000	1.000	0.400	1.000	0.500
201895_at	ARAF	1.000	NA	NA	0.200	1.000	0.400	1.000
202424_at	MAP2K2	1.000	1.000	NA	1.000	0.333	1.000	1.000
202670_at	MAP2K1	0.182	1.000	1.000	0.111	1.000	0.667	1.000
202738_s_at	PHKB	0.667	1.000	1.000	1.000	1.000	1.000	1.000
204357_s_at	LIMK1	0.667	1.000	1.000	0.500	0.167	NA	1.000
206044_s_at	BRAF /// KIAA1549	1.000	0.286	1.000	1.000	1.000	0.667	1.000
206984_s_at	RIT2	1.000	0.500	1.000	1.000	1.000	0.333	0.182
209882_at	RIT1	0.286	1.000	0.400	0.333	0.200	0.500	0.333
209953_s_at	CDC37	1.000	NA	1.000	0.667	1.000	1.000	0.667
211936_at	HSPA5	0.667	0.800	1.000	1.000	1.000	1.000	0.182
212046_x_at	MAPK3	0.200	NA	0.667	0.167	0.500	1.000	1.000
235252_at	KSR1	0.400	NA	1.000	0.010	1.000	NA	0.400
214487_s_at	RAP2A /// RAP2B	0.154	1.000	0.267	0.667	0.010	0.667	0.020
218669_at	RAP2C	0.667	0.167	0.172	0.167	0.667	1.000	1.000
225414_at	RNF149	1.000	1.000	1.000	0.125	1.000	1.000	0.500
226391_at	BRAF	1.000	NA	0.333	0.333	0.667	0.667	1.000

Tests were performed across 15 drugs and BRAF-related genes.
